# Conformational signatures in β-arrestin2 reveal natural biased agonism at a G-protein-coupled receptor

**DOI:** 10.1038/s42003-018-0134-3

**Published:** 2018-09-03

**Authors:** Arfaxad Reyes-Alcaraz, Yoo-Na Lee, Seongsik Yun, Jong-Ik Hwang, Jae Young Seong

**Affiliations:** Graduate School of Medicine, Korea University, Seoul, 02841 Republic of Korea

## Abstract

Discovery of biased ligands and receptor mutants allows characterization of G-protein- and β-arrestin-mediated signaling mechanisms of G-protein-coupled receptors (GPCRs). However, the structural mechanisms underlying biased agonism remain unclear for many GPCRs. We show that while Galanin induces the activation of the galanin receptor 2 (Galr2) that leads to a robust stimulation toward Gαq-protein and β-arrestin1/2, an alternative ligand Spexin and its analog have biased agonism toward G-protein signaling relative to Galanin. We used intramolecular fluorescein arsenical hairpin bioluminescence resonance energy transfer-based biosensors of β-arrestin2 combined with NanoBit technology to measure β-arrestin2–Galr2 interactions in real-time living systems. We found that Spexin and Galanin induce specific active conformations of Galr2, which may lead to different internalization rates of the receptor as well as different signaling outputs. This work represents an additional pharmacological evidence of endogenous G-protein-biased agonism at a GPCR.

## Abstract (web summary)

Arfaxad Reyes-Alcaraz et al. report that galanin induces robust signaling mediated by β-arrestin1/2 and Gαq, whereas an alternative ligand spexin prefers the Gαq-protein signaling pathway. This study provides mechanistic insights into how endogenous ligands can generate biased signaling outputs.

## Introduction

**Fig. 1:**
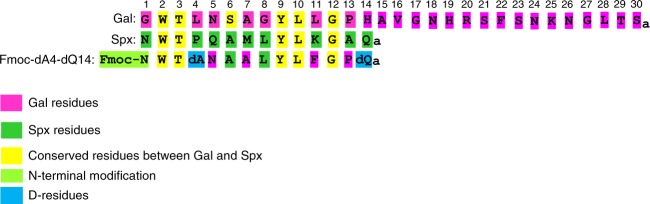
Amino acid sequence alignment of human Galanin, Spexin, and the Galr2-selective agonist Fmoc-dA4-dQ14. Galanin and Spexin residues are shown in pink and green, respectively. Conserved amino acids are highlighted in yellow. N-terminus modifications and d-amino acids are marked in red and blue, respectively

G-protein-coupled receptors (GPCRs) are the largest family of cell surface receptors that communicate extracellular stimuli to the cell interior^1^. Biased ligands are of particular interest because they offer the possibility to design a novel class of therapeutic agents^2^. In some GPCRs, biased ligands that preferentially activate G-protein-dependent signaling have therapeutic potential^3^. Biochemical and biophysical study results suggest that different ligands can induce or stabilize, or both, subsets of the multiple active conformations of a receptor^4–7^. Most descriptions of biased agonism have focused on the actions of synthetic ligands^4^. However, the existence of multiple endogenous ligands targeting the same GPCR suggests the potential for “endogenous” biased agonism. Biased agonism by endogenous peptides has been found in some GPCRs^8,9^. These findings may partly explain the apparent redundancy of such systems. However, the potential for bias within the endogenous galanin receptor system has not been investigated. Galanin receptors are activated by two peptide ligands, Spexin and Galanin^10^. These ligands have different sequences, structures, and molecular weights (Fig. 1). Galanin has high affinities toward galanin receptor 1 (Galr1) and Galr2, but low affinity toward galanin receptor 3 (Galr3). Spexin activates Galr2 and Galr3 with high affinities but does not activate Galr1^10^. Thus, both Galanin and Spexin stimulate Galr2 and are generally localized in brain regions with Galr2 expression^11,12^. Galanin and Spexin participate in numerous neurophysiological processes mediated by this receptor (e.g., feeding behavior, reproduction, neuroprotection, learning, memory, cardiovascular, renal function, and nociception)^11–25^. The actions of Galanin and Spexin are often opposed, particularly in appetite behavior and reproduction. For example, Galanin administration to mice results in a food intake increase^26,27^. Spexin suppresses appetite in goldfish^15^, and leads to weight loss in diet-induced obese rodents^13^. Thus, Galanin appears to be an orexigenic, and Spexin is an anorexigenic. Administration of galanin-like peptide, a Galanin paralog, stimulates luteinizing hormone (LH) secretion in the rat;^28^ Spexin administration attenuates LH secretion in the goldfish^29^. These opposing effects are likely at least partly due to Galr subtype-specific signaling pathways. Alternatively, biased agonism between Galanin and Spexin might be associated with control of Galr2-mediated physiologic processes.

In the current study, we investigated the differential signaling of Galr2 induced by two structurally different endogenous ligands and the Spexin-based long acting Galr2 agonist, Fmoc-dA4-dQ14^30^. The bias agonism between each signaling pathway was quantified to obtain unique biased agonism profiles for these ligands. The results indicate that Spexin and Fmoc-dA4-dQ14 exert biased agonism toward G_αq_-protein signaling. Galanin induces robust signaling toward G-protein and β-arrestin1/2. We aimed to determine the molecular mechanisms underlying biased agonism at Galr2, and to establish a link between different receptor/β-arrestin conformations and selective functional outcomes. We used a panel of fluorescein arsenical hairpin (FlasH) bioluminescence resonance energy transfer (BRET)-based biosensors to monitor conformational changes in β-arrestin2^31,32^. We also used two structural complementation assays to accurately monitor β-arrestin recruitment and receptor trafficking. We found that Galanin, Spexin, and Fmoc-dA4-dQ14 impose distinctive β-arrestin2 conformational hallmarks that reflect differential signaling of the receptor–β-arrestin2 complex. The innate properties of each peptide are revealed as changes in β-arrestin2 conformation. Our results indicate how different endogenous ligands can use Galr2 for different purposes.

## Results

### Differential β-arrestin recruitment at Galr2

**Fig. 2:**
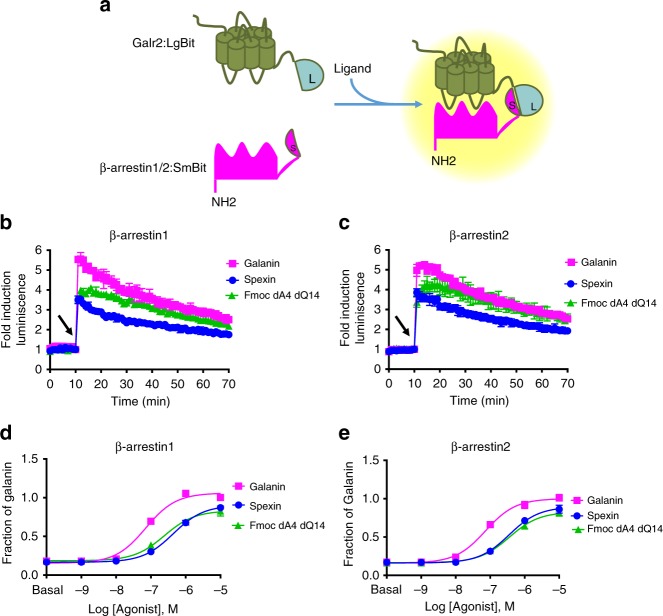
The effect of different endogenous ligands in β-arrestin1/2 recruitment. **a** Schematic representation of the structural complementation assay used to monitor β-arrestin1/2–Galr2 interactions. Kinetic traces of β-arrestin1 (**b**) and β-arrestin2 (**c**) recruitment after Galr2 activation with 1 μM of three different agonists. Dose–response curves for recruitment of β-arrestin1 (**d**), and β-arrestin2 (**e**). Luminescence signal intensity obtained at 5 min after agonist stimulation was measured. The data were normalized to the maximal response induced by Galanin and were fit using a three-parameter model of agonism (Eq. 1). The results are expressed as mean ± s.e.m. of three experiments performed in triplicate; each triplicate was averaged before calculating the s.e.m. The arrows indicate the time at which the cells were treated with the different ligands

We examined the potential for Spexin, Galanin, and Fmoc-dA4-dQ14 to differentially recruit β-arrestin isoforms at Galr2. The β-arrestin recruitment assays were developed based on a structural complementation assay using NanoBit technology^33^. Briefly, NanoBit technology is a system that basically consists of Nano luciferase (Nluc), which is a small protein that catalyzes a bright luminescent reaction. To create a complementation system, Nluc was separated into two subunits that individually produce very little enzymatic activity. The Large Bit (LgBit) showed an excellent structural stability, while the Small Bit (SmBit) was characterized by its low affinity to the LgBit^33^. Because of this low affinity for each other, these two fragments can bind when they are in close proximity. The interaction between the LgBit and the SmBit can be driven by the interactions of the fusion partners, allowing them to study protein–protein interaction in live cells. As the interaction is reversible, the association and dissociation can be monitored. There is no lag time in the loss of luminescence, and so the immediate detection allows for accurate assessment of temporal dynamics^33^. The results indicated that the Galr2–β-arrestin1 and Galr2–β-arrestin2 interactions were transient, and registered a maximum of intensity immediately after ligand stimulation (Fig. 2). To accurately monitor β-arrestin recruitment, four plasmid combinations between LgBit and SmBit with the fusion partners Galr2 and β-arrestins were screened. The plasmid combination with the highest luminescence signal was chosen for further studies (Fig. 2a, Supplementary Fig. 1). Using data points at 5 min after ligand stimulation, we obtained dose–response curves that corresponded to β-arrestin1/2 recruitment (Fig. 2d, e, Supplementary Fig. 2). The results indicate that Spexin and Fmoc-dA4-dQ14 are likely partial agonists in the recruitment of both β-arrestin isoforms.

### Differential interaction between β-arrestin2 and clathrin

**Fig. 3:**
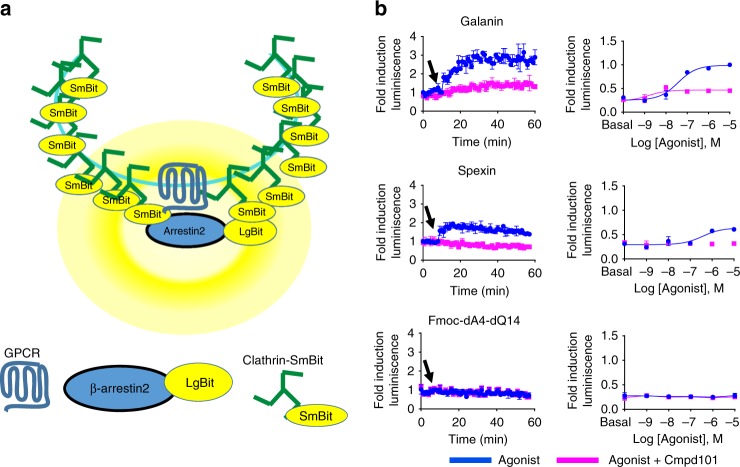
Differential β-arrestin2–clathrin interactions induced by endogenous ligands. **a** Schematic representation of the experimental design used to monitor agonist-promoted luminescence after Galr2 stimulation. **b** Time course and dose–response of β-arrestin2–clathrin interactions by 10 μM Galanin, Spexin, and Fmoc-dA4-dQ14 in the presence or absence of 30 μM of Cmpd101, a GRK2/3 inhibitor. The arrows indicate the time when the cells were treated with different ligands. For dose–response curve, the data obtained at 5 min after agonist stimulation were normalized to the maximal response induced by Galanin. The results are expressed as mean ± s.e.m. of three experiments performed in triplicate. Each triplicate was averaged before calculating the s.e.m.

The interaction between β-arrestin2 and clathrin induced by the activation of Galr2 was examined using the NanoBit technology. We measured real-time association of the protein complex Galr2–β-arrestin2–clathrin. We constructed β-arrestin2 and clathrin subunit A tagged with LgBit or SmBit at the C or N termini and screened eight different plasmid combinations, out of which only one combination (β-arrestin2–LgBit:SmBit–clathrin) responded to ligand stimulation (Fig. 3, Supplementary Fig. 3). Before ligand stimulation, Galr2, β-arrestin2, and clathrin were not in close proximity and so the basal luminescence was very low. Upon Galr2 activation, redistribution of the receptor and the association of these three proteins resulted in an increase in luminescence (Fig. 3a). A time course experiment was initially performed to determine the time at which the maximal increase in luminescence occurred following agonist stimulation. All ligands except for Fmoc-dA4-dQ14 induced different fold increase in luminescence immediately after ligand treatment (Fig. 3b). This result indicated that rapid receptor redistribution occurred. Dose–response curves revealed that Galanin displayed the highest response in formation of Galr2-β–arrestin2–clathrin complex. Spexin exhibited lower potency and efficacy than Galanin in this assay. Fmoc-dA4-dQ14 did not induce β-arrestin2–clathrin association at all different concentrations. Galanin- and Spexin-induced increases in luminescence were attenuated by pretreatment of cmpd101, a G-protein-coupled receptor kinase 2/3 (GRK2/3) inhibitor, indicating contribution of GRK2/3 in the formation of Galr2–β-arrestin2–clathrin complex (Fig. 3).

### Differential Galr2 internalization

**Fig. 4:**
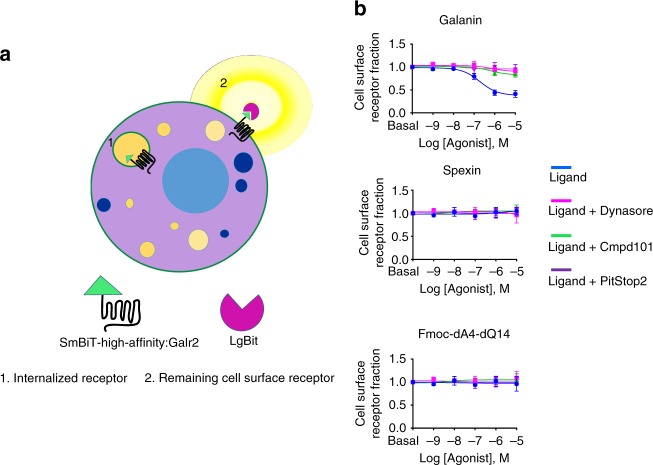
Internalization of Galr2. **a** Schematic representation of the internalization assay. Only the SmBiT-High-affinity:Galr2 remained in the cell surface after ligand stimulation can bind to LgBit to produce the luminescent signal. **b** HEK293 cells transiently transfected with 0.5 ng/well of Galr2 containing the SmBiT-High-affinity tag at the N termini. Before ligand stimulation, cells were pretreated with 30 μM Cmpd101 (a GRK2/3 inhibitor) for 30 min, 25 μM PitStop 2 (a Clathrin inhibitor) for 15 min, and 80 μM Dynasore (a Dynamin inhibitor) for 40 min. Cells were then treated with different concentrations of Galanin, Spexin, and Fmoc-dA4-dQ14 for 30 min. Remaining cell surface receptors were determined by measuring the luminescent signal produced by the binding between LgBiT and the Galr2-SmBiT-High affinity. Each data point represents mean ± s. e. m. of two independent experiments performed in triplicate

As Spexin and Fmoc-dA4-dQ14 exhibited a partial agonism in β-arrestin recruitment, we then examined Galr2 internalization using HiBiT technology^34,35^. The HiBiT protein tagging system, like NanoBiT technology, consists of two subunits: the large fragment (LgBiT) with 156 amino acids and the small fragment of Nluc (SmBiT-High-affinity) with only 11 amino acids that has a very high affinity to LgBit. To quantify Galr2 at the cell surface, cells expressing Galr2 tagged with the SmBiT-High-affinity were treated with cell-impermeable LgBiT along with the Nluc substrate furimazine. SmBiT-High-affinity-Galr2 on the cell surface binds to LgBiT which produces a luminescent signal, while SmBiT-High-affinity-Galr2 inside the cells cannot bind to LgBiT (Fig. 4a). Pretreatment of Galanin for 30 min significantly decreased the luminescent signal in a dose-dependent manner (Fig. 4b), indicating that Galanin is able to induce internalization of the receptor. This internalization of Galr2 was partially blocked by the GRK2/3 inhibitor cmpd101 and completely blocked by dynamin and clathrin inhibitors, suggesting an important role of these proteins during the regulation of Galr2 internalization. In contrast to Galanin, we did not observe the substantial decrease of luminescent signal in Spexin- and Fmoc-dA-dQ14-pretreated cells, indicating that these agonists marginally induce internalization of the receptor.

### Differential G-protein signaling and Erk1/2 phosphorylation

**Fig. 5:**
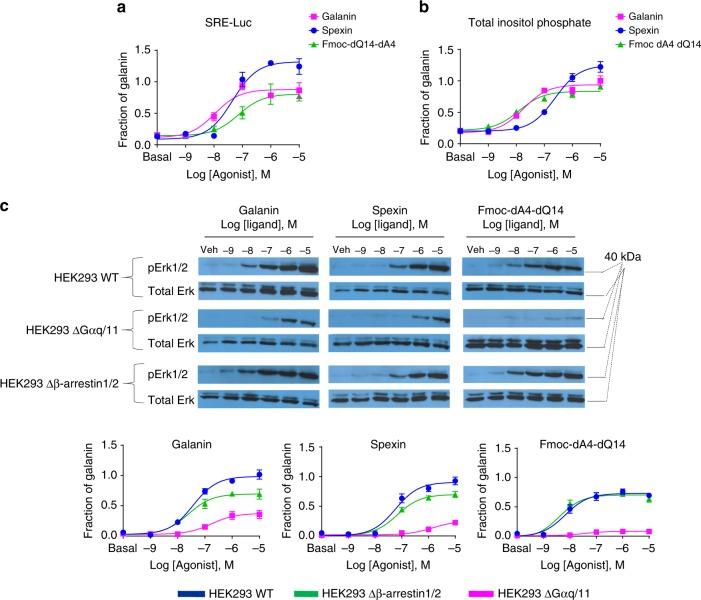
Galr2 agonists display different G_αq_-protein-dependent signaling and pErk1/2. The ability of increasing concentrations of agonists to induce **a** SRE-Luc activity and **b** total inositol phosphate production. **c** Representative western blot showing ligand-induced phosphorylation of Erk1/2 from wild-type (HEK293 WT), G_αq/11_-, and β-arrestin1/2-knockout HEK293 cells expressing Galr2 (original blots are presented in Supplementary Figure 4). pErk1/2 and total Erk levels were determined at 5 min after stimulation of different concentrations of three ligands. Western blot band intensities corresponding to each concentration were quantified using Image studio ^TM^ Software. The data shown in the dose–response curve were normalized to the maximal response induced by Galanin and were fit using a three-parameter model (Eq. 1). Data are the mean ± s.e.m. values of two independent experiments

Galr2 is primarily coupled to the G_αq_-protein which activates the phospholipase C/protein kinase C pathway for inositol phosphate production^36^. We measured G-protein activation in HEK293 cells transiently expressing Galr2, using the serum response element-luciferase (SRE-Luc) gene reporter assay^10,37^ and for a more specific monitoring of G_αq_-protein activity we also measured total inositol phosphate production. Spexin had substantially higher efficacy in both G-protein-dependent assays; Fmoc-dA-dQ14 displayed total inositol phosphate production similar to Galanin (Fig. 5b). However, some differences were observed between SRE-Luc activity and total inositol phosphate production in the rank order of potency of the tested ligands. These differences suggest that in our SRE-Luc system might be activated by an additional signaling pathway at Galr2 not exclusively dependent of G_αq_-protein. Phosphorylation of extracellular signal-regulated protein kinases 1 and 2 (pErk1/2) is either G-protein or β-arrestin dependent. To distinguish G_αq_-protein- and β-arrestin1/2-dependent Erk1/2 signaling, we examined ligand-stimulated Erk1/2 phosphorylation in three different HEK293 cell lines, wild type, G_αq/11_-knockout, and β-arrestin1/2-knockout cells. All three ligands induced high levels of Erk1/2 phosphorylation dose-dependently in wild-type HEK293 cells. This agonist-stimulated Erk1/2 phosphorylation was largely attenuated in G_αq/11_-knockout cells but slightly decreased in β-arrestin1/2-knockout cells, indicating that agonist-induced Erk1/2 phosphorylation particularly in the case of Spexin and Fmoc-dA4-dQ14 is likely mainly attributable to the G-protein-dependent signaling pathway (Fig. 5c, Supplementary Fig. 4).

### Detection of biased agonism at Galr2

**Fig. 6:**
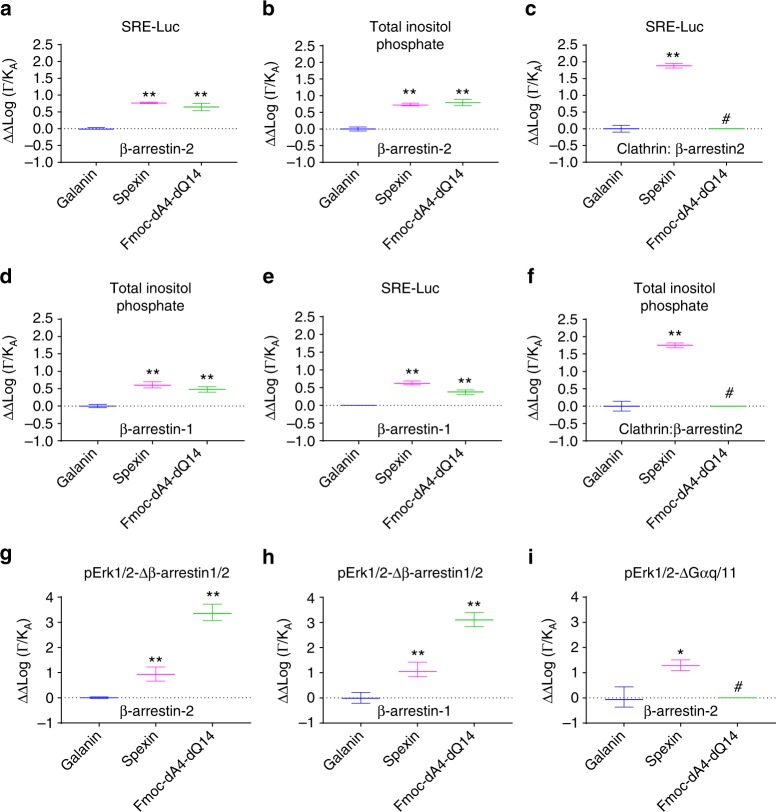
Biased factors across different β-arrestins and G-protein signaling pathways at Galr2. The dose–response curves for G-protein- and β-arrestin-dependent signaling were analyzed using an operational model of agonism (Eq. 2) to obtain transduction coefficients (Log(*τ*/*K*_A_)). These coefficients were normalized to the corresponding value obtained for the reference agonist Galanin (ΔLog(*τ*/*K*_A_)). The normalized values obtained for one agonist at two different pathways were subtracted to obtain bias factor values (ΔΔLog(*τ*/*K*_A_). These bias factor values for different agonists **a** SRE-Luc activity and β-arrestin2 recruitment, **b** total inositol phosphate production and β-arrestin2 recruitment, **c** SRE-Luc activity and Galr2 internalization, **d** total inositol phosphate and β-arrestin1 recruitment, **e** SRE-Luc activity and β-arrestin1 recruitment, **f** total inositol phosphate production and β-arrestin2:clathrin interaction, **g** pErk1/2-Δβ-arrestin1/2 and β-arrestin2, **h** pErk1/2-Δβ-arrestin1/2 and β-arrestin1, and **i** pErk1/2-ΔG_αq/11_ and β-arrestin1 in reference to Galanin are represented in the graphs. The results are expressed as the mean ± s.e.m. values of three independent experiments. *P < 0.05; **P < 0.005 statistically significant differences versus Galanin as determined by a one-way analysis of variance (ANOVA). ^#^Means not determined. **a** *P* = 0.0083, **b** *P* = 0.0023, **c** *P* = 0.0023, **d** *P* = 0.0043, **e** *P* = 0.0002, **f** *P* < 0.0001, **g** *P* < .0.0001, **h** *P* < 0.0001, **i** *P* = 0.0081

To determine whether differential signaling at Galr2 (Figs. 2 and 5) may be translated into biased agonism, we used the operational model of agonism (Eq. 2) to calculate the logarithm of the transduction coefficient (log(*τ*/*K*_A_)) for each agonist at each pathway (Table 1)^4^. We used Galanin as a reference ligand during all experiments. The log(*τ*/*K*_A_) values were normalized to those for Galanin at each pathway (Δlog(*τ*/*K*_A_), Table 2); they were compared across different signaling pathways to obtain ΔΔlog(*τ*/*K*_A_) or bias factor values (Table 2). The bias factor values are graphically presented in Fig. 6. The results revealed the biased agonism among Galanin, Spexin, and Fmoc-dA4-dQ14. Spexin and Fmoc-dA4-dQ14 had biased agonism toward G_αq_-protein-dependent signaling over β-arrestins and β-arrestin2–clathrin interactions (Fig. 6). In concordance with this result, we found that Spexin tended to display biased agonism toward SRE-Luc and total inositol phosphate production, compared with β-arrestin1/2. We also calculated bias factors for G-protein-dependent pErk1/2 and β-arrestins. In the case of Spexin and its analog we observed biased signaling toward G_αq_-dependent pErk1/2 over the two β-arrestin isoforms. In the case of Spexin biased agonism was also detected toward β-arrestin-dependent pErk1/2 over β-arrestin2 recruitment, suggesting that β-arrestin1 might also contribute to pErk1/2 signaling. According to the information obtained from Galr2 internalization (Fig. 4) we also detected biased agonism in this regard, where Galanin efficiently induced internalization in a dose-dependent manner, but a very different behavior was observed when Galr2 was activated by Spexin or Fmoc-dA4-dQ14 where Galr2 internalization was not detected (Fig. 4b).

**Table 1:** Log(*τ*/*K*_A_) and ΔLog(*τ*/*K*_A_) values determined for Galr2 agonists at different signaling pathways

Log(τ/K_A_) (ΔLog(τ/K_A_))*	Galanin	Spexin	Fmoc-dA4-dQ14
SRE-Luc	4.80±0.007 (0.00 ± 0.007)	4.95±0.04 (0.09 ± 0.02)	4.60±0.04^a^ (0.16 ± 0.04)^a^
Total inositol phosphate	4.20 ± 0.03 (0.00 ± 0.02)	4.40 ± 0.03^a^ (0.05 ± 0.01)	4.18 ± 0.02 (−0.05 ± 0.02)
β-Arrestin2	7.20 ± 0.02 (0.00 ± 0.02)	6.40 ± 0.03^b^ (−0.70 ± 0.03)^b^	6.32 ± 0.10^a^ (−0.8 ± 0.10)^a^
β-Arrestin1	7.18 ± 0.01 (0.00 ± 0.01)	6.60 ± 0.08^a^ (−0.60 ± 0.01)^b^	6.62 ± 0.07^a^ (−0.55 ± 0.07)^a^
Clathrin:β-arrestin2	8.00 ± 0.18 (0.00 ± 0.20)	6.22 ± 0.10^a^ (−1.80 ± 0.10)^a^	ND (ND)
G_αq_-dependent-pErk1/2	6.90 ± 0.22 (0.00 ± .22)	7.47 ± 0.21 (0.57 ± 0.22)	ND (ND)
β-Arrestin1/2 dependent-pErk1/2	6.42 ± 0.41 (0.00 ± 0.40)	7.15 ± 0.14(0.73 ± 0.13)	ND(ND)

Parenthesis indicate ΔLog(*τ*/*K*_A_) values. Log(*τ*/*K*_A_) and ΔLog(*τ*/*K*_A_) values were determined for Galr2 agonists for total inositol phosphate production, SRE-Luc activity, pERK1/2, β-arrestin1/2, and internalization using Galanin as reference agonist for experiments performed in all assays. Results are expressed as mean ± s.e.m. values of three experiments performed in triplicate. Each triplicate was averaged before calculating the s.e.m.

*ND* not determined

^a^*P* < 0.05

^b^*P* ≤ 0.005 indicates significant differences from Galanin as determined by Student’s unpaired two-tailed *t*-test

**Table 2:** Quantification of biased agonism at Galr2 using the operational model of agonism

ΔΔLog(*τ*/*K*_A_)	Galanin	Spexin	Fmoc-dA4-dQ14
SRE-Luc/β-arrestin2	0.00 ± 0.02	0.77 ± 0.02^b^	0.64 ± 0.10^b^
Total inositol phosphate/β-arrestin2	0.00 ± 0.05	0.73 ± 0.09^b^	0.75 ± 0.12^b^
SRE-Luc/β-arrestin1	0.00 ± 0.002	0.63 ± 0.06^b^	0.37 ± 0.06^b^
Total inositol phosphate/β-arrestin1	0.00 ± 0.03	0.60 ± 0.09^b^	0.50 ± 0.06^b^
SRE-Luc/clathrin:β-arrestin2	0.00 ± 0.17	1.88 ± 0.07^b^	ND
Total inositol phosphate/clathrin:β-arrestin2	0.00 ± 0.19	1.84 ± 0.12^b^	ND
G_αq_-dependent-pErk1/2/β-arrestin2	0.00 ± 0.04	0.88 ± 0.30^b^	3.35 ± 0.35^b^
G_αq_-dependent-pErk1/2/β-arrestin1	0.00 ± 0.22	1.10 ± 0.29^b^	3.00 ± 0.28^b^
β-Arrestin-dependent-pErk1/2/β-arrestin2	0.00 ± 0.38	1.40 ± 0.16^a^	ND

Bias factors were determined for Galr2 agonists between total inositol phosphate production, SRE-Luc activity, and β-arrestin1/2 as well as clathrin:β-arrestin2 interactions, using Galanin as a reference agonist from experiments performed in all assays. Results are expressed as mean ± s.e.m. values of three experiments performed in triplicate. Each triplicate was averaged before calculating the s.e.m.

*ND* not determined

^a^*P* < 0.05

^b^*P* < 0.005 indicates significant differences from Galanin at two different pathways for a particular ligand, as determined by the results of Student’s unpaired two-tailed *t*-test

### Distinct conformational changes in β-arrestin2 upon activation of Galr2

**Fig. 7:**
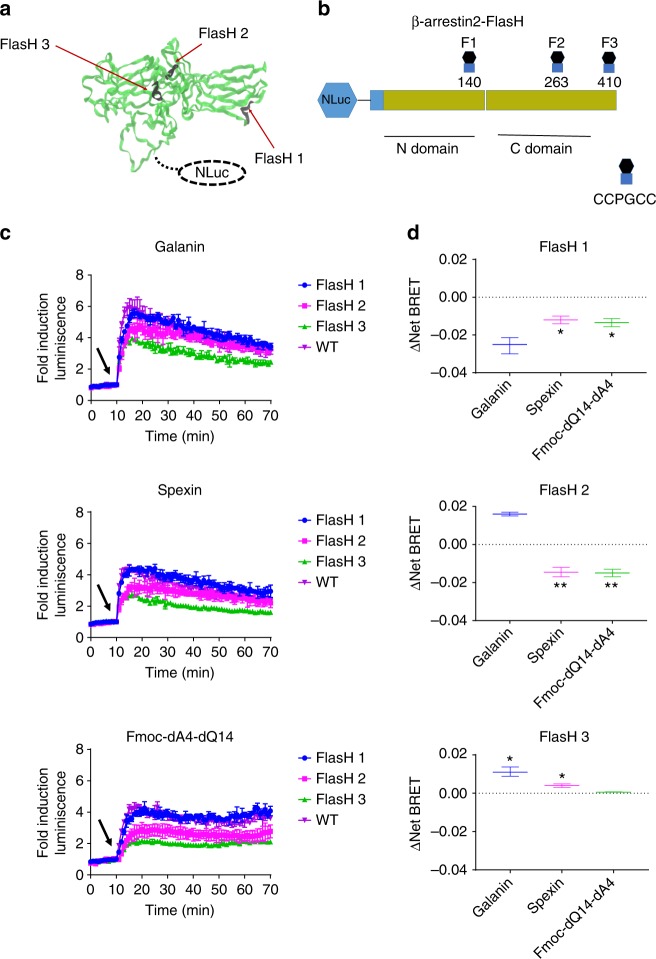
Design of Nluc–β-arrestin2–FlasH BRET conformational biosensors and β-arrestin2 conformational hallmarks imposed by different conformations of Galr2. **a**, **b** Three Nluc–β-arrestin2–FlasH BRET conformational biosensors were constructed by inserting the amino acid motif CCPGCC after amino acid residues 140, 263, and 410 of human β-arrestin2; the location of each motif is shown in relation to the globular N and C domains of β-arrestin2. **c** Functionality assay based on NanoBit technology demonstrating ligand-dependent recruitment of β-arrestin2-FlasH1-3 to human Galr2. The arrows indicate the time for treatment with the corresponding agonist. **d** Nluc–β-arrestin2–FlasH1-3 “conformational hallmarks” of β-arrestin2 by binding to Galr2. The bar graphs depict mean ± s.e.m. values of independent biological replicates (*n* = 3). *P < 0.05, **P < 0.005, statistically significant differences versus Galanin-stimulated control as determined by a one-way analysis of variance (ANOVA). *P*_FlasH1_ = 0.0284, *P*_FlasH2_ = 0.0039, *P*_FlasH3_ = 0.0357

To investigate the effects of GPCR activation on the dynamics of β-arrestin2 conformation, we developed a series of FlasH BRET conformational biosensors^31,32^ by inserting the six-amino-acid motif, CCPGCC, immediately after the amino acid residue 140 (FlasH1), 263 (FlasH2), and 410 (FlasH3), respectively, into the human β-arrestin2 (Fig. 7a, b). Due to its characteristics (e.g., small size, stable, and bright and sustained luminescence)^33^, we covalently linked the Nluc to the N terminus of β-arrestin2 during probe design. Each probe (Nluc–β-arrestin2–FlasH1, 2, and 3) was designed to measure BRET between Nluc and a fluorescein arsenical acceptor located at one of three positions along β-arrestin2. We hypothesized that observing changes in BRET efficiency from multiple sites would produce a β-arrestin2 conformational hallmark characteristic for each ligand^31^. We used a structural complementation assay based on NanoBit technology to test whether the insertion of the FlasH motif would have an effect on β-arrestin2 recruitment. All three ligands were able to recruit these β-arrestin2–FlasH constructs to induce luminescence signals, although some signals were lower than that of wild-type β-arrestin2 (Fig. 7c). Like wild-type β-arrestin2 recruitment assay, Galanin among the ligands induced the maximal response in recruitment of three β-arrestin2–FlasH constructs. We then tested whether Galr2 activation produces an intramolecular Nluc–β-arrestin2–FlasH BRET signal upon recruitment to untagged Galr2 (Supplementary Fig. 5). The agonists elicited differential changes in the Nluc–β-arrestin2–FlasH BRET signal (ΔNet BRET). Spexin and Fmoc-dA4-dQ14 displayed ΔNet BRET values in each probe significantly different from those observed in Galanin (Fig. 7d). This result indicates that different conformations in β-arrestin2 are imposed by distinct structural rearrangements at Galr2, probably leading to different signaling outputs.

### Differential ligand–receptor dissociation kinetics

**Fig. 8:**
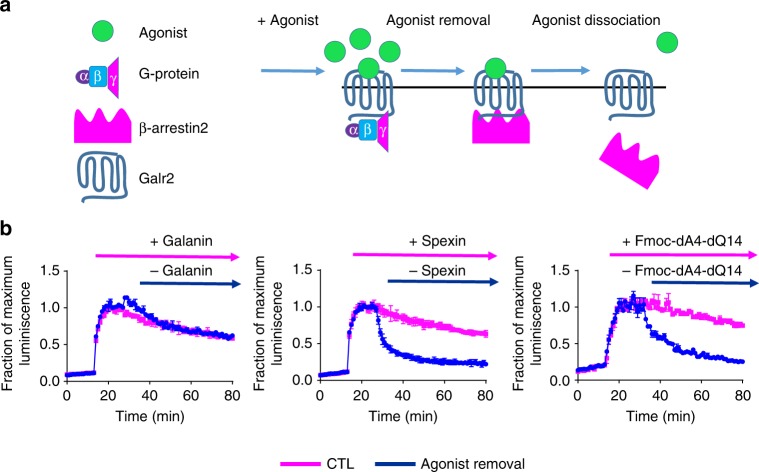
Reversal assay approach used to estimate agonist-receptor dissociation rate. **a** Schematic representation describing that the recruitment of β-arrestin2 to Galr2 is dependent on ligand–receptor dissociation after ligand removal. **b** Cells were incubated with 10 μM agonist to permit a stable β-arrestin2 response. Ligand removal was performed by simply changing the medium containing no ligand and then immediately continue measuring luminescence. Samples with no ligand removal were used as a control. In Spexin- and Fmoc-dA-dQ14-treated cells, ligand removal caused a rapid decrease in luminescence signal back to basal levels. In Galanin-treated cells, decrease in luminescence signal after ligand removal was not such rapid. The results are expressed as the mean ± s.e.m. values of two independent experiments

A recent article suggested that kinetic context of ligand towards the receptor significantly contributes to biased signaling^4^. As the interactions between Galr2 and β-arrestins are ligand dependent, we hypothesized that the ligand dissociation from the receptor can be indirectly determined by measuring the kinetics of the dissociation of the Galr2–β-arrestin2 complex after removal of agonist by infinite dilution (Fig. 8a). The decay of the luminescent signal may reflect dissociation rate of ligand from Galr2 as the β-arrestin2 response returns to basal levels in washout experiments. In Galanin-treated cells, the luminescent signals slowly decreased after removal of agonist, indicating that Galanin remains bound to the receptor–β-arrestin2 complex for a relatively long time with a half-life (*t*_*1*/2_) >49.5 ± 5 min. In contrast Spexin and Fmoc-dA4-dQ14 appear to be rapidly dissociated from the receptor as the luminescent signals rapidly returned to baseline levels after ligand washing, registering *t*_1/2 _= 3.35 ± 0.7 min and 12 ± 2 min for Spexin and Fmoc-dA4-dQ14, respectively (Fig. 8b). Differences in ligand–receptor dissociation rate among Galanin, Spexin, and Fmoc-dA4-dQ14 contributed, at least in part, to different β-arrestin2 recruitment followed by different rates of receptor internalization, leading to a biased agonism by Spexin and Fmoc-dA4-dQ14 relative to Galanin.

## Discussion

We devised several strategies to accurately measure β-arrestin recruitment and Galr2 internalization in live cells. We designed structural complementation assays based on NanoBit and HiBiT technologies. The differential β-arrestin recruitment, G_αq_-protein-dependent signaling, and the major differences in ligand–receptor internalization suggest that Galanin and Spexin stabilize different receptor conformations that can be translated into endogenous biased agonism. Biased factors between β-arrestin1/2 and G_αq_-protein-dependent signaling revealed that in HEK293 cells, Spexin and Fmoc-dA4-dQ14 use the G_αq_-protein as a principal effector to produce signaling through Galr2. This result may suggest that each endogenous ligand has a specific physiological function for which biased agonism is used as a mechanism to regulate a diversity of biological events where Galr2 is involved.

Spexin is an additional endogenous ligand for Galr2^10^. Therefore, these structurally distinct ligands might induce different conformations on the receptor and lead to different structural rearrangements on β-arrestins. To determine whether Galr2 can adopt distinct conformations induced by different endogenous ligands, we monitored the Net ΔBRET signal upon stimulation of Galr2 by Galanin, Spexin, or Fmoc-dA4-dQ14 using a panel of FlasH BRET conformational biosensors based on human β-arrestin2. Our hypothesis was that distinct conformations of Galr2 impose different structural rearrangements on β-arrestin2 during its recruitment. Stimulation of Galr2 with Spexin or Fmoc-dA4-dQ14 resulted in ΔNet BRET changes in the three FlasH biosensors that were significantly different from those with Galanin. Because ΔNet BRET results from changes in the distance or orientation, or both, of the donor and acceptor molecules, significant differences in ΔNet BRET in the same biosensor may arise from distinct positional changes between Nluc (donor) and FlasH (acceptor)^31,32^. In other words, significant differences in the ΔNet BRET indicate different conformations of β-arrestin2 imposed by different structural conformations in the receptor. A ΔNet BRET increase versus a decrease indicates major conformational differences at β-arrestin2. This result suggests that a ΔNet BRET decrease in Flash2 might be closely related to Galr2 conformations with lower affinity toward β-arrestin2, determining biased agonism toward G-protein-dependent signaling. Spexin and Fmoc-dA4-dQ14 had very similar signaling profiles and we did not detect Galr2 internalization induced by Spexin and Fmoc-dA4-dQ14. In addition, the ΔNet BRET values were very similar between Spexin and Fmoc-dA4-dQ14 except for the FlasH3 conformational biosensor, suggesting that conformational changes at the C terminus of β-arrestin2 might be associated with its dual functions (e.g., receptor internalization, desensitization, or both).

Most current studies of biased agonism have been restricted to use synthetic ligands. Evidence of natural bias at some GPCRs has been revealed^8,9,38^. In this study, we found structural and pharmacological evidence that biased agonism might be a natural mechanism for GPCRs with redundancy of ligands by stabilizing different receptor conformations. Because Spexin and Galanin have wide amino acid sequence diversity, we hypothesized that these two endogenous ligands can exert differential signaling through the same receptor, Galr2.

Recently, the impact of the kinetic context on the quantification of biased agonism has been documented^39^. In order to have a better picture of the G-protein biased agonism at Galr2, we designed a strategy to indirectly measure the residence time of the ligand at the receptor by a reversal dissociation assay. According to our results, Spexin and Fmoc-dA4-dQ14 act as fast dissociation agonists with a residence time significantly shorter than Galanin. Thus, we proposed that Spexin and Fmoc-dA4-dQ14 display G-protein biased signaling by stabilizing a Galr2 conformation that does not retain the agonist for a long time, being dissociated easily after G-protein activation and β-arrestin recruitment. In contrast, Galanin is able to induce an active conformation on the receptor that retains Galanin for a relatively long time which may allow Galr2 to continue producing different signals by interacting with a wide variety of cytosolic partners along the time. The potency of Galanin in internalization assay is about 10-fold lower than that observed in other assays such as β-arrestin recruitment. It raises a possibility that we could not observe Galr2 internalization induced by Spexin and Fmoc-dA4-dQ14 because the signal might be less amplified than other β-arrestin-dependent assays. However, the ligand–receptor dissociation kinetics revealed that Spexin and its analog act as fast dissociation agonists relative to Galanin, suggesting that in some GPCRs the ligand residence time can determine whether the receptor can be internalized or not, depending on the time that the ligand remains bound to receptor. According to our model, Spexin and Fmoc-dA4-dQ14 dissociate from the receptor immediately after G_αq_ activation or at the beginning of β-arrestin recruitment with no sufficient time left to proceed with the internalization process.

It would also be interesting to confirm this hypothesis using a real ligand-binding kinetics assay, since there is a possibility that the dissociation of the ligand from the receptor may not occur exactly at the same time as the dissociation of β-arrestin2. This possibility could not be excluded, using our approach. We consider that ligand residence time is likely a critical point to explain at least partly a possible mechanism of biased signaling on a specific GPCR.

Studies have revealed the role of Galr2 in synaptic plasticity^11^, in neuropsychiatric disorders (e.g., anxiety and depression)^21^, and its neuroprotective effects in neurodegenerative diseases (e.g., multiple sclerosis)^12^. The study of the benefits of signaling in this receptor is of vital importance. Galanin and its receptors were discovered almost 40 years ago^40^. However, their β-arrestin signaling remains uncharacterized. The possibility of endogenous biased agonism, especially at Galr2 and Galr3, is more evident since the discovery that Spexin can also efficiently activate these two receptors. To quantify the differential signaling at Galr2, we used a systematic method based on the calculation of bias factors and used Galanin as a reference. Examination of bias across multiple pathways revealed the complex nature of biased agonism at Galr2. It revealed another level of complexity of bias that extends beyond differential activation of G-proteins and β-arrestin recruitment. Current study may expand choice of options to develop Galr2 agonists (developing Galanin-based agonists or Spexin-based biased agonists) with fewer side effects in vivo.

## Methods

The ability of Galr2 to produce differential signaling in response to Galanin, Spexin, and Fmoc-dA4-dQ14 was assessed in HEK293 cells expressing human Galr2. Quantification of bias between each pathway was performed using Galanin as a reference ligand. All incubations for the different signaling assays were performed in serum-free medium to prevent peptide degradation.

### Materials

Cell culture medium and cell culture additives were from Life Technologies.

### Chemicals and peptides

All chemicals were obtained from Sigma-Aldrich (St. Louis, MO, USA) unless otherwise stated. The restriction enzymes were obtained from New England Bio Labs (Ipswich, MA, USA). All ligand peptides were synthesized by AnyGen (Gwangju, Korea). The synthesized peptide purity was greater than 98% as determined by high-performance liquid chromatography analysis. All peptides were dissolved in dimethyl sulfoxide and then diluted in media to the desired working concentrations.

### NanoBit technology

The NanoBit starter kit containing the plasmids and the necessary reagents for the development of the structural complementation assays used in this study was a gift from Promega Company (Madison, Wisconsin, USA).

### β-Arrestin1/2 recruitment using NanoBit technology

HEK293 cells were obtained from the American Type Culture Collection (ATCC, CRL-1573; Manassas, VA, USA). They were maintained in Dulbecco’s modified Eagle’s medium (DMEM) supplemented with 10% fetal bovine serum (FBS), 100 U/ml penicillin G, and 100 μg/ml streptomycin (Invitrogen; Carlsbad, CA, USA). During the experiments, the cells were tested for mycoplasma using a Universal Mycoplasma Detection Kit (ATCC). At 1 day before transfection, the cells were seeded in 96-well plates at a density of 2.5 × 10^4^ cells per well. A mixture containing 50 ng β-arrestin construct containing the LgBit or SmBit and 50 ng Galr2 containing one of the two domains of Nluc and 0.2 μl Lipofectamine 2000 (Invitrogen) was prepared and added to each well. We tested four β-arrestin-Galr2 spatial orientations. The one with the highest signal was chosen for further experiments to increase the assay sensitivity. At 24 h post transfection, the medium was aspirated and replaced with 100 μl OPTIMEM (Life Technologies, Grand Island, NY, USA) at room temperature. After a 10-min incubation, 25 μl substrate (furimazine) was added and once every minute subsequent luminescence measurements were taken for 10 min for signal stabilization. A total of 10 μl ligand was then added to each well and luminescence measurements were recorded immediately and once every minute for 1 h (Synergy 2 Multi-Mode Microplate Reader BioTek, Winooski, VT, USA).

### Detection of β-arrestin2–clathrin interactions

One day before transfection, cells were seeded in 96-well plates at a density of 2.5 × 10^4^ cells per well. A mixture containing 35 ng β-arrestin construct containing the LgBit or SmBit, 35 ng clathrin subunit A containing one of the two domains of Nluc and 30 ng untagged receptor, and 0.2 μl Lipofectamine 2000 (Invitrogen) was prepared and added to each well. We followed the assay manufacturer’s instructions and tested eight β-arrestin-clathrin plasmid combinations. In only one plasmid combination we were able to observe luminescent signal and it was chosen for further experiments. At 24 h post transfection, medium was aspirated and replaced with 100 μl OPTIMEM at room temperature. After a 10-min incubation, 25 μl substrate (furimazine) was added and the luminescence was monitored once every minute for 10 min. Then, 10 μl ligand was added to each well and the luminescence values were recorded immediately and once every minute for 1 h (Synergy 2 Multi-Mode Microplate Reader BioTek).

### Internalization assay based on HiBiT technology

Internalization assays were performed using the Nano-Glo HiBiT extracellular detection system acquired from Promega Corporation (Cat. No. N2421). The day before transfection, HEK293 cells were seeded in 96-well plates at a density of 2.5 × 10^4^ cells per well. The following day, cells were transfected with a mixture prepared containing 0.5 ng of SmBiT-high-affinity-Galr2 construct and 0.2 μl Lipofectamine 2000 (Invitrogen) added to each well. At 24 h post transfection, wells allocated for inhibitor pretreatment cells were pretreated with 30 μM of GRK2/3 inhibitor cmpd101 from Tocris Bioscience (Cat. No. 5642) for 30 min, 25 μM of clathrin inhibitor PitStop 2 from Abcam (Cat. No. ab120687) for 15 min, and 80 μM dynamin inhibitor Dynasore from Abcam (Cat. No. ab120192) for 40 min, and then treated using different agonist concentrations for 30 min, immediately after 100 μl of Nano-Glo HiBiT extracellular reagent (1 μl of LgBiT protein + 2 μl Substrate + 97 μl of Nano-Glo HiBiT buffer) was added and left to equilibrate for 4 min at room temperature without mixing. Then luminescence values were recorded immediately (Synergy 2 Multi-Mode Microplate Reader BioTek). Luminescent values were normalized in reference to cells treated with vehicle only.

### Cell transfection and luciferase assay

One day before transfection, cells were seeded in 48-well plates at a density of 2.5 × 10^4^ cells per well. A mixture containing 75 ng SRE-Luc reporter construct, 75 ng expression plasmid, and 0.3 μl Lipofectamine 2000 (Invitrogen) was prepared and added to each well, according to the manufacturer’s instructions. The cells were then maintained in serum-free DMEM for 16–18 h before exposure to the ligands. Approximately 48 h after transfection, the cells were treated with the respective ligands for 6 h. The cells were then lysed using 100 μl lysis buffer. The luciferase activity in 50 μl cell extract was determined using a luciferase assay system, following the standard protocol for the Synergy 2 Multi-Mode Microplate Reader Biotek.

### Total inositol phosphate production

Galr2 activation was monitored using measurement of total inositol phosphate (IP + IP2 + IP3) production in HEK293 cells expressing human Galr2. For this assay, cells were seeded in 12-well plates at a density of 2.5 × 10^5^ cells per well. The next day, the cells were transiently transfected with 1 μg/well plasmid containing human Galr2, using the Lipofectamine 2000 (Invitrogen) transfection reagent. The day after transfection, the cells were incubated in M199 medium containing 1% FBS, 1% l-glutamine, 1% penicillin/streptomycin, and 1 μCi/well myo-^3^H inositol for 20 h. After a 30-min incubation in buffer A (140 mM NaCl, 20 mM Hepes, 4 mM KCl, 8 mM d-glucose, 1 mM MgCl_2_, 1 mM CaCl_2_, 1 mg/ml free fatty acid bovine serum albumin, and 10 mM LiCl at pH 7.2), the cells were exposed to the agonist for 40 min at 37 °C. The medium was removed, and addition of 1 ml 10 mM cold formic acid to each well was used to terminate the reaction. The plates were stored at 4 °C for 30 min, and the extracts were then transferred into 6 ml plastic tubes containing 500 μl AG1–8X anion exchange columns (BIO-RAD, Hercules, CA, USA). The tubes were gently vortexed, and the supernatants were removed using aspiration. Two washes with 1 ml distilled water were then performed, followed by two washes with 60 mM ammonium formate/5 mM sodium tetraborate. Tritiated inositol was separated from the column using elution with 1 ml of 1 M ammonium formate/0.1 M formic acid; 800 μl of the elution was taken from each tube and transferred into 6 ml scintillation vials. Then, 2 ml scintillation cocktail solution (Ultima GoldTM, Perkin Elmer, Waltham MA, USA) was added to each sample. The radioactivity of the samples was measured using a TRI carb 3100TR liquid scintillation analyzer (Packard).

### β-Arrestin2 conformational biosensors

The design and development of the FlasH BRET conformational biosensors was based on information previously described^31^. Briefly, a set of three Nluc–β-arrestin2–FlasH BRET biosensors were constructed using a modified overlap extension PCR method and inserting a complementary DNA (cDNA) sequence encoding the amino acid motif, CCPGCC, immediately after the amino acids 140, 263, and 410 of human β-arrestin2^31,41^. To generate each construct, three PCR steps were performed using the primer sets shown in Supplementary Table 1. The initial step was the generation of the construct human β-arrestin2 tagged at the N terminus with Nluc via fusion of two PCR fragments using the primer pairs NlucBamHIF–NlucR and BA2F–BA2EcoRIR. One PCR product contained a *Bam*HI restriction site at the 5′ end and the motif of the C terminus of Nluc at the 3′ end. The other product contained the complementary Nluc sequence at the 5′ end and an *Eco*RI restriction site at the 3′ end. A second PCR step was used to fuse the two fragments using these two PCR products as mega primers. Finally, the fusion product was amplified in a third PCR step using the primers NlucBamHIF and BA2EcoRIR. The resultant chimeric PCR product full-length Nluc–β-arrestin2 PCR product was digested using *Bam*HI and *Eco*RI and cloned into the pcDNA3.1+ expression plasmid. For the generation of the Nluc–β-arrestin2–FlasH constructs, the chimeric construct Nluc–β-arrestin2 was used as a template; three PCR steps used the primer sets shown in Supplementary Table 1. During the first step, we generated two PCR fragments using the primer pairs NlucBamHIF–FlasHR and FlasHF–BA2EcoRIR. One PCR product contained a *Bam*HI restriction site at the 5′ end and the CCPGCC FlasH motif at the 3′ end. The other product contained the complementary FlasH sequence at the 5′ end and an *Eco*RI restriction site at the 3′ end. These two PCR products were used as mega primers during a second PCR step used to fuse the two fragments. The resultant full-length β-arrestin2 PCR product containing the FlasH motif was further amplified during a third PCR step. The resulting PCR product was digested using *Bam*HI and *Eco*RI and ligated into the parent Nluc–β-arrestin2 construct to obtain the Nluc–β-arrestin2–FlasH1–3 expression constructs^31^. Each construct was corroborated by sequencing.

### Intramolecular BRET at β-arrestin2 conformational biosensors

Intramolecular BRET at β-arrestin2 conformational biosensors was measured based on the steps and procedures previously described^31^. Briefly, HEK293 cells were seeded in 6-well plates using cell density of 6 × 10^5^ cells per well. The next day, the cells were co-transfected with 1.5 μg plasmid DNA encoding the receptor of interest and 0.1 μg DNA encoding one Nluc–β-arrestin2–FlasH construct using Lipofectamine 2000 reagent. The cells were detached using citrate saline buffer (135 mM KCl, 15 mM Sodium citrate) 48 h after transfection and the cells were collected using centrifugation, and the pellets were resuspended in 600 μl Opti-MEM®. TC-FlasH II In-Cell Tetracystein detection reagent was added to a 2.5 μM final concentration and the cells were incubated at room temperature for 1 h. After incubation the cells were washed using 1× BAL buffer from the TC-FlasH kit, resuspended in OPTIMEM and placed in white-wall clear-bottom 96-well plates at a density of 1 × 10^5^ cells per well. Background and total TC-FlasH fluorescence were read on a microplate reader (Fluoroskan Ascent™ FL) with 485 nm excitation and 538 nm emission filters. All ligand treatments were performed at maximum ligand concentrations (10 μM). The cells were treated with agonist and furimazine at the same time. After 2 min, three consecutive readings of Nano Luciferase (470 nm) and TC-FlasH (535 nm) emissions were obtained, and the BRET ratio (emission TC-FlasH/emission Nluc) was calculated using the Fluoroskan. The ΔNet change in intramolecular BRET ratio for each of the three Nluc–β-arrestin2–FlasH biosensors was calculated by background-subtracting the BRET ratio measured for the cells in the same plate treated with Opti-MEM as a vehicle.

### pErk1/2 assay

pErk1/2 was measured using western blot assay. HEK293 G_αq/11_ and β-arrestin1/2-knockout cells were a kind gift from Professor Asuka Inoue, Tohoku University. The cells were seeded into 6-well plates at a density of 6 × 10^5^ cells per well. The next day, they were transfected using 2 μg/well of plasmid containing human Galr2 cDNA and Lipofectamine 2000, according to the manufacturer’s instructions. Before assay, the cells were washed with phosphate-buffered saline and incubated in serum-free DMEM overnight. Dose–response experiments were performed for each ligand at 37 °C. Stimulation of the cells was terminated after 5 min of agonist stimulation by removing the medium, and the addition of 250 μl cold lysis buffer to each well. Immunoblots of phospho-Erk1/2 were obtained using 8 μg of total protein and p44/42 MAPK Erk1/2 antibody (Cell Signaling Technology Inc, catalog no. 4370 S); horseradish peroxidase-conjugated goat anti-rabbit IgG was used as a secondary antibody (Thermo Fisher Scientific). The proteins were visualized using enhanced chemiluminescence (GE Health Care). Image Studio ^TM^ Software was used for the pErk1/2 band density analysis.

### Reversal assay for dissociation kinetics

One day before transfection, the cells were seeded in 96-well plates at a density of 2.5 × 10^4^ cells per well. A mixture containing 50 ng β-arrestin2 construct containing the SmBit and 50 ng Galr2 containing the LgBiT at the C termini plus 0.2 μl Lipofectamine 2000 (Invitrogen) was prepared and added to each well. At 24 h post transfection, the medium was aspirated and replaced with 100 μl OPTIMEM and incubated at room temperature. After a 10-min incubation, 25 μl substrate (furimazine) was added, and once every minute subsequent luminescence measurements were taken for 10 min for signal stabilization. A total of 10 μl ligand was then added to each well and luminescence measurements were recorded immediately and once every minute during 10 min for signal stabilization. After that time wells allocated for ligand removal, ligand containing medium was replaced for medium containing only furimazine substrate and no ligand. Immediately continued recording the time course luminescence values once every minute during 1 h.

### Data analysis

The results were analyzed using the Prism 7 application (Graph Pad Software Inc., San Diego, CA). Dose–response curves were fitted using the following three-parameter equation:

1 $${\mathrm{Response}} = {\mathrm{Bottom}} + \frac{{{\mathrm{Top}} - {\mathrm{Bottom}}}}{{1 + 10^{\left( {{\mathrm{log}}{\mathrm{EC}}_{50} - \log \left[ A \right]} \right)}}}$$

where Bottom and Top are the lower and upper plateaus, respectively, of the concentration–response curve, [*A*] is the molar concentration of the agonist, and EC_50_ is the molar concentration of agonist required to generate a response halfway between the top and the bottom.

To calculate biased factors and compare agonist profiles, the dose–response data were fit to the following form of the operational model of agonism:^42^

2 $${Y} = {\mathrm{Basal}} + \frac{{\left( {{E}_{m} - {\mathrm{basal}}} \right)\left( {\frac{{\mathrm{\tau }}}{{{K}_{A}}}} \right)^{\mathrm{n}}[{A}]^{\mathrm{n}}}}{{[{A}]^{\mathrm{n}}\left( {\frac{{\mathrm{\tau }}}{{{K}_{A}}}} \right)^{\mathrm{n}} + \left( {1 + \frac{{[{A}}]}{{{K}_{A}}}} \right)^{\mathrm{n}}}}$$

where *E*_m_ is the maximal possible response of the system, Basal is the basal level of response, *K*_A_ represents the equilibrium dissociation constant of the agonist (*A*), and *τ* is an index of the signaling efficacy of the agonist that is defined as *R*_T_/*K*_E_. *R*_T_ is the total number of receptors, *K*_E_ is the coupling efficiency of each agonist-occupied receptor, and *n* is the slope of the transducer function that links occupancy to response. One assumption of the analysis is that the transduction machinery used for a given cellular pathway are the same for all agonists, such that the *E*_m_ and transducer slope (*n*) are shared between agonists. The data for all compounds for each pathway were fit globally, to determine values of *K*_A_ and *τ*. Biased agonism was quantified as previously described^42^. Briefly, to exclude the effect of cell-dependent and assay-dependent effects on the observed agonism at each pathway, the log(*τ*/*K*_A_) value of a reference agonist, Galanin, is subtracted from the log(*τ*/*K*_A_) value of the agonists of interest to yield ΔΔlog(*τ*/*K*_A_). The relative bias can then be calculated for each agonist at the two different signaling pathways by subtracting the Δlog(*τ*/*K*_A_) of one pathway from the other to give a ΔΔlog(*τ*/*K*_A_) value, which is a measure of bias. A lack of biased agonism will result in values of ΔΔlog(*τ*/*K*_A_) not significantly different from 0 between pathways^43^. All transduction ratios (log(*τ*/*K*_A_)) parameters were estimated as logarithms. All results were expressed as mean ± s.e.m. values. We performed a Brown–Forsythe test (Graph Pad Prism 7.0) to assure ourselves of equal variances between the compared parameters.

## Electronic supplementary material

Supplementary informaton

## Data Availability

Data set corresponding to differential signaling at Galr2 is available for download at Dryad (https://datadryad.org/review?doi=doi:10.5061/dryad.vh13401). Additional data that support the findings of this study are available in supplementary information and from the corresponding author upon request.

## References

[1] Pierce KL, Premont RT, Lefkowitz RJ (2007). Seven-transmembrane receptors. Nat. Rev. Mol. Cell Biol..

[2] Violin JD, Lefkowitz RJ (2007). B-arrestin-biased ligands at seven-transmembrane receptors. Trends Pharmacol. Sci..

[3] Raehal KM, Walker JK, Bohn LM (2005). Morphine side effects in beta-arrestin 2 knockout mice. J. Pharmacol. Exp. Ther..

[4] Herenbrink CK (2016). The role of kinetic context in apparent biased agonism at GPCRs. Nat. Commun..

[5] Saulière A (2012). Deciphering biased-agonism complexity reveals a new active AT1 receptor entity. Nat. Chem. Biol..

[6] Swaminath G (2005). Probing the β2 adrenoceptor binding site with catechol reveals differences in binding and activation by agonists and partial agonists. J. Biol. Chem..

[7] Vilardaga JP, Steinmeyer R, Harms GS, Lohse MJ (2005). Molecular basis of inverse agonism in a G protein-coupled receptor. Nat. Chem. Biol..

[8] Thompson GL (2015). Biased agonism of endogenous opiod peptides at the μ-opioid receptor. Mol. Pharmacol..

[9] Rajagopal S (2013). Biased agonism as a mechanism for differential signaling by chemokine receptors. J. Biol. Chem..

[10] Kim DK (2014). Coevolution of the Spexin/Galanin/Kisspeptin family: Spexin activates galanin receptor type II and III. Endocrinology.

[11] Badie-Mahdavi H, Lu X, Behrens MM, Bartafi T (2005). Role of galanin receptor 1 and galanin receptor 2 activation in synaptic plasticity associated with 3’, 5’ -cyclic AMP response element-binding protein phosphorylation in the dentate gyrus: studies with a galanin receptor 2 agonist and galanin receptor 1 knock out mice. Neurosicience.

[12] Elliot-Hunt CR, Pope RJ, Vanderplank P, Wynick D (2007). Activation of the galanin receptor 2 (GalR2) protects the hippocampus from neuronal damage. J. Neurochem..

[13] Walewski JL (2014). Spexin is a novel human peptide that reduces adipocyte uptake of long chain fatty acids and causes weight loss in rodents with diet-induced obesity. Obesity.

[14] Toll L (2012). Peptides derived from the prohormone proNPQ/Spexin are potent central modulators of cardiovascular and renal function and nociception. FASEB J..

[15] Wong MK (2013). Goldfish spexin: solution structure and novel function as a satiety factor in feeding control. Am. J. Physiol. Endocrinol. Metab..

[16] Mazarati AM (2004). Galanin and galanin receptors in epilepsy. Neuropeptides.

[17] Blackshear A (2007). Intracerebroventricular administration of galanin or galanin receptor subtype agonist M617 induces c-Fos activation in central amygdala and dorsomedial hypothalamus. Peptides.

[18] Wynick D, Thompsom SW, McMahon SB (2001). The role of galanin as a multi-functional neuropeptide in the nervous system. Curr. Opin. Phrmacol..

[19] Elliot-Hunt CR (2011). Endogenous galanin protects mouse hippocampal neurons against amyloid toxicity in vitro via activation of galanin receptor 2. J. Alzheirmers Dis..

[20] Hobson SA, Holmes FE, Kerr NC, Pope RJ, Wynick D (2006). Mice deficient for galanin receptor 2 have decreased neurite outgrwth from adult sensory neurons and impaired pain-like behaviour. J. Neurochem..

[21] Bailey KR, Pavlova MN, Rohde AD, Hohmann JG, Crawley JN (2007). Galanin receptor subtype 2 (GalR2) null mutant mice display an anxiogenic-like phenotype specific to the elevated plus-maze. Pharmacol. Biochem. Behav..

[22] Lang R, Gundlach AL, Kofler B (2007). The galanin peptide familiy: receptor pharmacology, pleitropic biological actions, and implications in health and disease. Pharmacol. Ther..

[23] Lundström L, Elmquist A, Bartfai T, Langel U (2005). Galanin and its receptors in neurological disorders. Neuromol. Med..

[24] Mitsukawa K, Lu X, Bartfai T (2008). Galanin, galanin receptors and drug targets. Cell. Mol. Life Sci..

[25] Hökfelt T, Tatemoto K (2008). Galanin--25 years with a multitalented neuropeptide. Cell. Mol. Life Sci..

[26] Rada P, Avena NM, Leibowitz SF, Hoebel BG (2004). Ethanol intake is increased by injection of galanin in the paraventricular nucleus and reduced by a galanin antagonist. Alcohol.

[27] Karatayev O, Baylan J, Leibowitz SF (2009). Increased intake of ethanol and dietary fat in galanin overexpressing mice. Alcohol.

[28] Castellano JM (2006). Effects of galanin-like peptide on luteinizing hormone secretion in the rat: sexually dimorphic responses and enhanced sensitivity at male puberty. Am. J. Physiol. Endocrinol. Metab..

[29] Liu Y (2013). A novel neuropeptide in suppressing luteinizing hormone release in goldfish, Carassius auratus. Mol. Cell. Endocrinol..

[30] Reyes-Alcaraz A (2016). Development of Spexin-based human galanin receptor type II specific agonists with increased stability in serum and anxiolytic effect in mice. Sci. Rep..

[31] Lee MH (2016). The conformational signature of β-arrestin2 predicts its trafficking and signaling functions. Nature.

[32] Nuber S (2016). Arrestin biosensors reveal a rapid, receptor dependent activation/deactivation cycle. Nature.

[33] Dixon AS (2016). NanoLuc complementation reporter optimized for accurate measurement of protein interactions in cells. ACS Chem. Biol..

[34] Schwinn MK (2018). CRISPR-mediated tagging of endogenous proteins with a luminescent peptide. ACS Chem. Biol..

[35] Oh-hashi K, Furuta E, Fujimura K, Hirata Y (2017). Application of a novel HiBiT peptide tag for monitoring ATF4 protein expression in Neuro2a cells. Biochem Biophys. Rep..

[36] Wang S, Hashemi T, Fried S, Clemmons AL, Hawes BE (1998). Differential intracellular signaling of the GalR1 and GalR2 galanin receptor subtypes. Biochemistry.

[37] Oh DY (2005). Membrane–proximal region of the carboxyl terminus of the gonadotropin-releasing hormone receptor (GnRHR) confers differential signal transduction between mammalian and nonmammalian GnRHRs. Mol. Endocrinol..

[38] Zidar DA (2011). Endogenous ligand bias by chemokines: implications at the front lines of infection and leukocyte trafficking. Endocr. Metab. Immune Disord. Drug Targets.

[39] Lane JR (2017). A kinetic view of GPCR allostery and biased agonism. Nat. Chem. Biol..

[40] Bedecs K, Berthold M, Bartfai T (1995). Galanin--10 years with a neuroendocrine peptide. Int. J. Biochem. Cell Biol..

[41] Wurch T, Lestienne F, Pauwels PJ (1998). A modified overlap extension PCR method to create chimeric genes in the absence of restriction enzymes. Biotechnol. Tech..

[42] Black JW, Leff P, Shankley NP, Wood J (1983). Operational models of pharmacological agonism. Proc. R. Soc. Lond. B.

[43] Kenakin T, Watson C, Muniz-Medina V, Christopoulos A, Novick SA (2012). A simple method for quantifying functional selectivity and agonist bias. ACS Chem. Neurosci..

