# Supplementary material for: Conformational signatures in β-arrestin2 reveal natural biased agonism at a G-protein-coupled receptor
Source: Commun Biol. 2018 Sep 3;1:128. doi: 10.1038/s42003-018-0134-3 (PMC6123711; doi:10.1038/s42003-018-0134-3)
Supplement: Supplementary file 1 — Supplementary informaton [file 42003_2018_134_MOESM1_ESM.pdf]

Supplementary Information

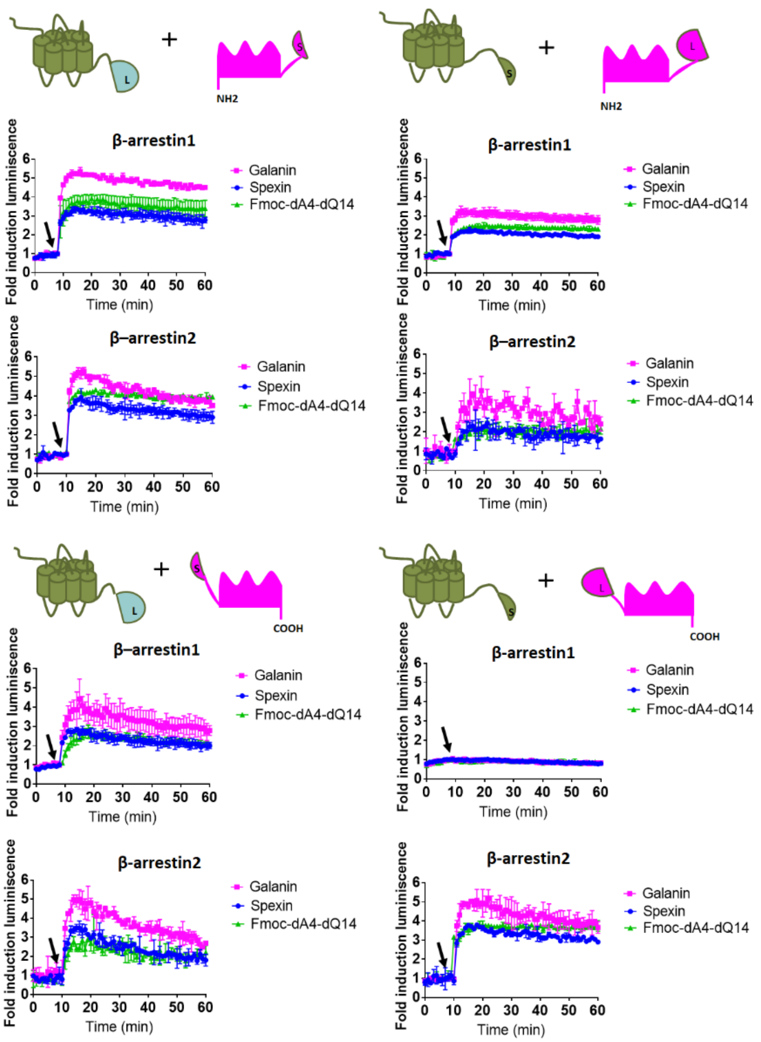

Supplementary Figure 1. Screening of β-arrestin1/2 recruitment kinetics. Four different plasmid combinations of β-arrestin1/2-Galr2 interactions containing the LgBit or SmBit at the C-terminal of Galr2 and β-arrestin1/2 containing LgBit or SmBit at the N- or C-terminal were screened in the presence of 1 μM of Spx, Gal, and Fmoc-dA4-dQ14. The arrows indicate the time when the cells were treated using the corresponding ligands. The data points are mean ± standard error of the mean (s.e.m.) values for three independent experiments performed in triplicate; each triplicate was averaged before calculating the s.e.m.

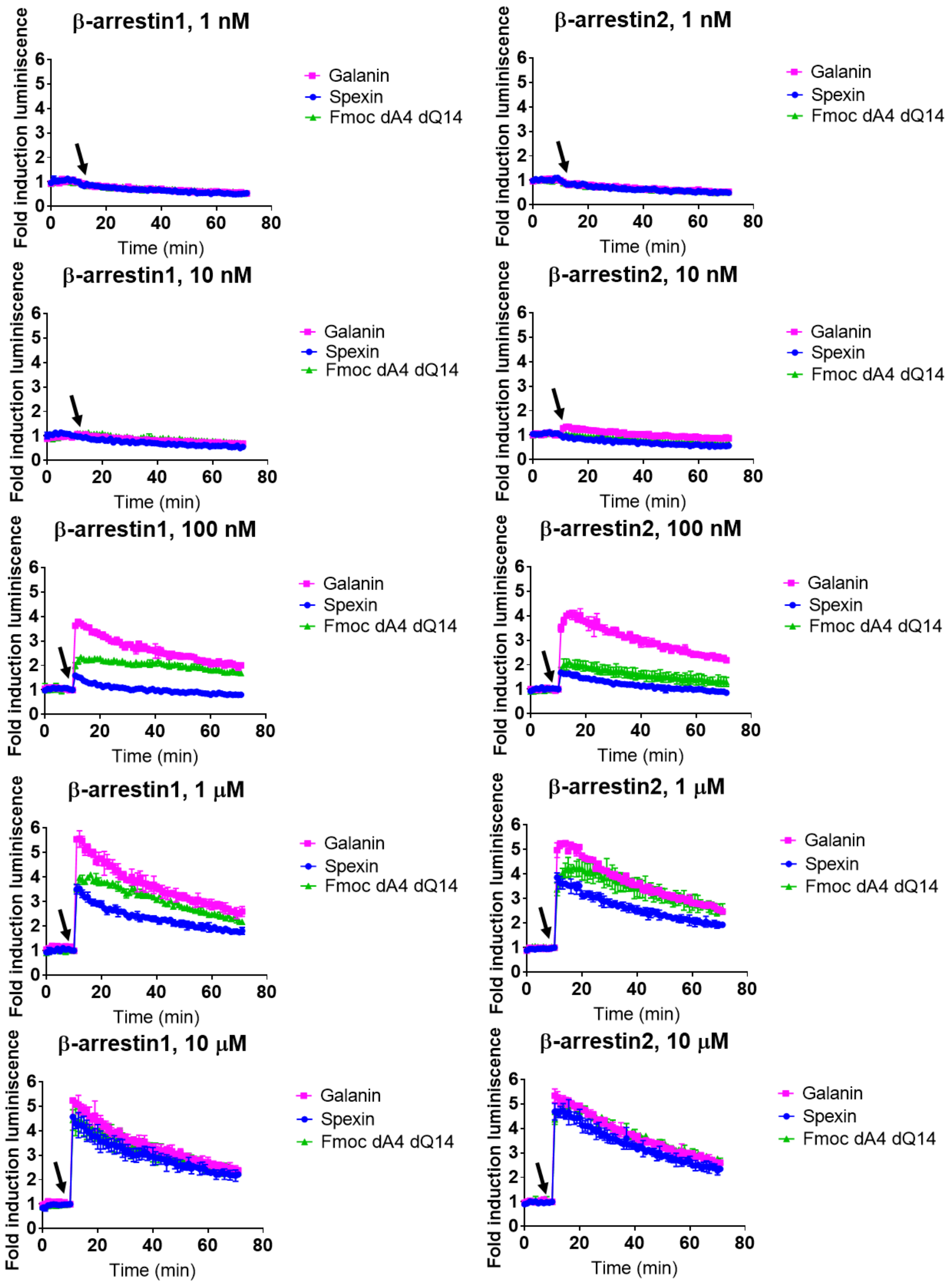

Supplementary Figure 2. Dose-response β-arrestin1/2 recruitment kinetics. Five different concentrations from 1 nM to 10 μM of each ligand were tested during the β-arrestin1/2 recruitment. The arrows indicate the time when the corresponding ligand was added. The data points are mean ± standard error (s.e.m.) values for three independent experiments performed in triplicate; each triplicate was averaged before calculating the s.e.m.

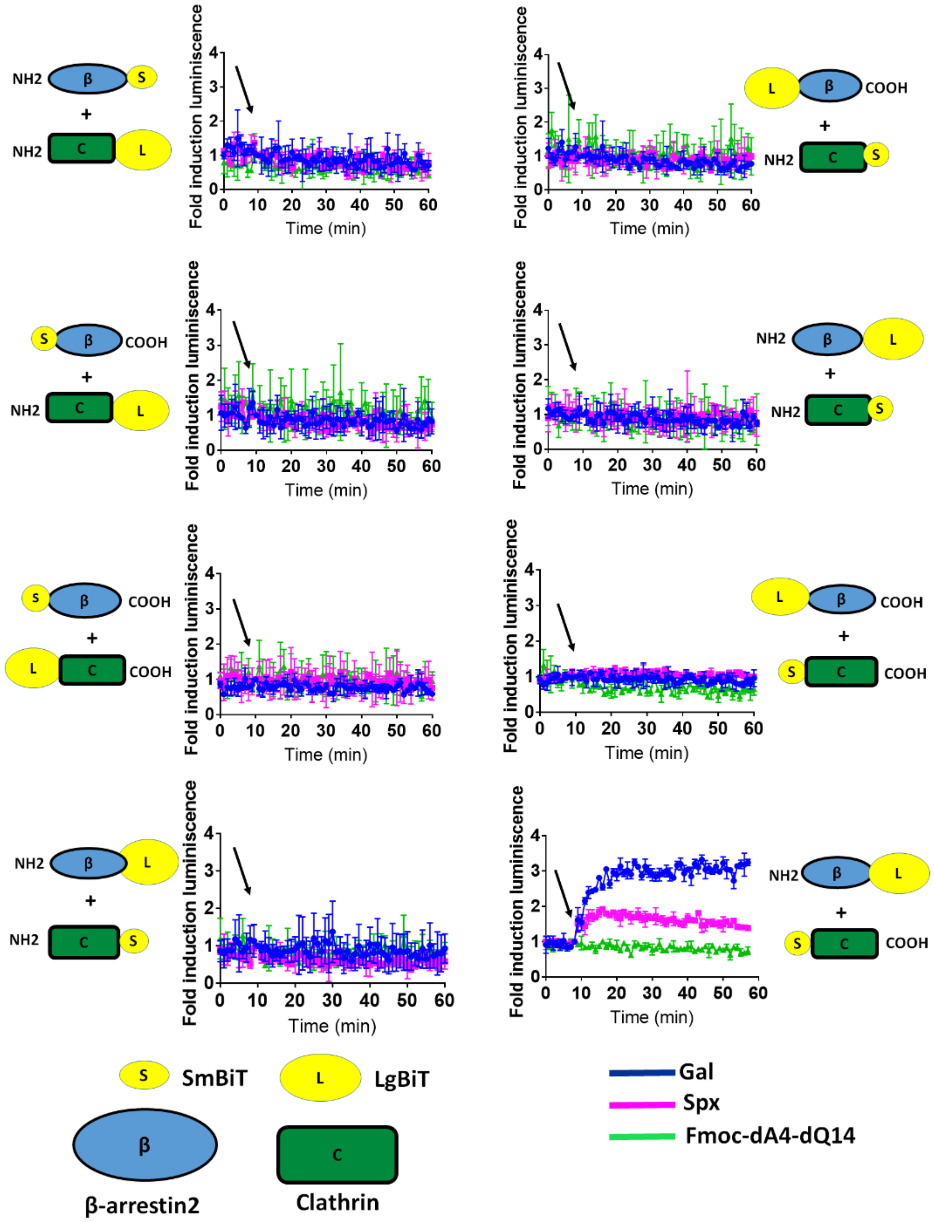

Supplementary Figure 3. Plasmid combination between clathrin and β-arrestin2 constructs containing LgBit and SmBit at N- or C-terminus in the presence of 10 µM of Gal, Spx, and Fmoc-dA4-dQ14. The arrows indicate the time when the cells were treated with the corresponding ligand. The data points are mean ± standard error (s.e.m.) values for three independent experiments performed in triplicate; each triplicate was averaged before calculating the s.e.m.

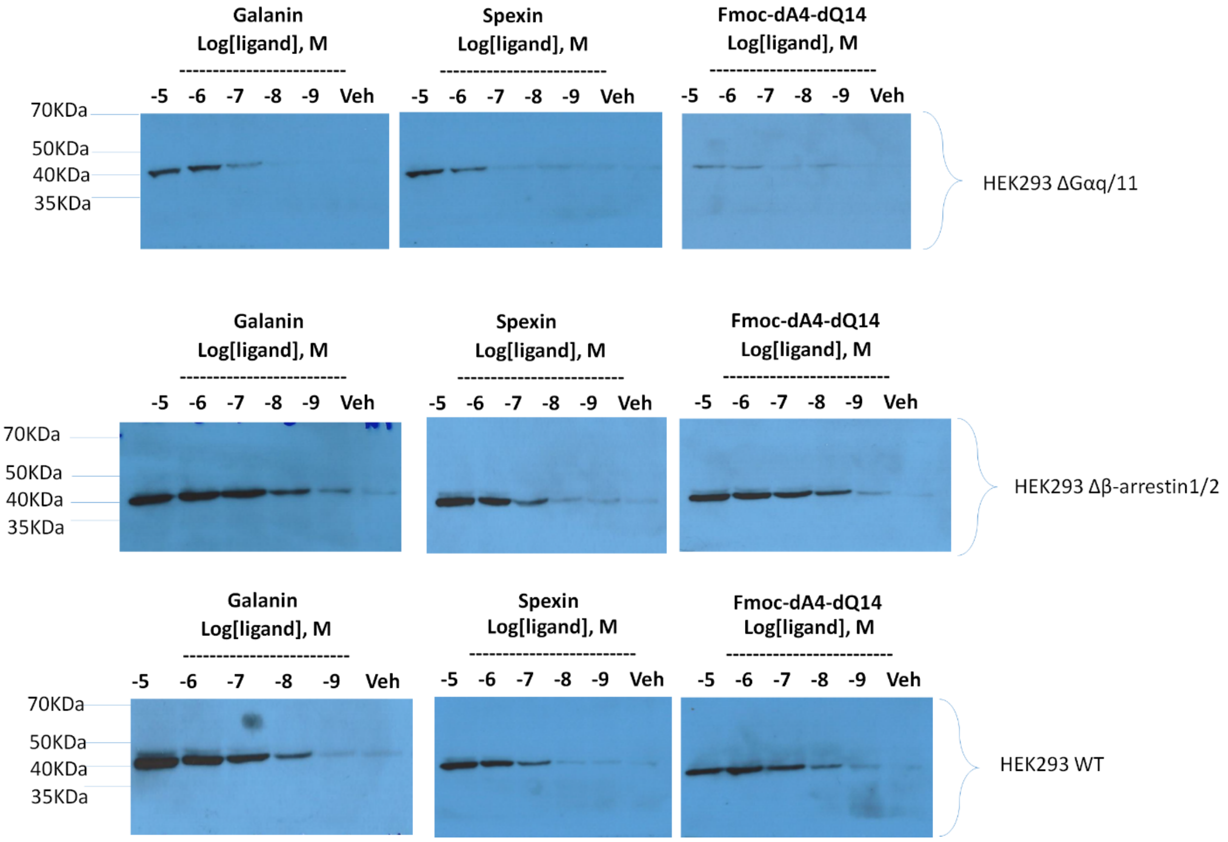

Supplementary Figure 4. Original Western blots showing ligand-induced phosporylation of Erk1/2 from wild type (HEK293 WT), $G_{\alpha q/11}$-, and β-arrestin1/2-knock out HEK293 cells expressing Galr2. pErk1/2 levels were determined at 5 min after stimulation of different concentrations of three ligands.

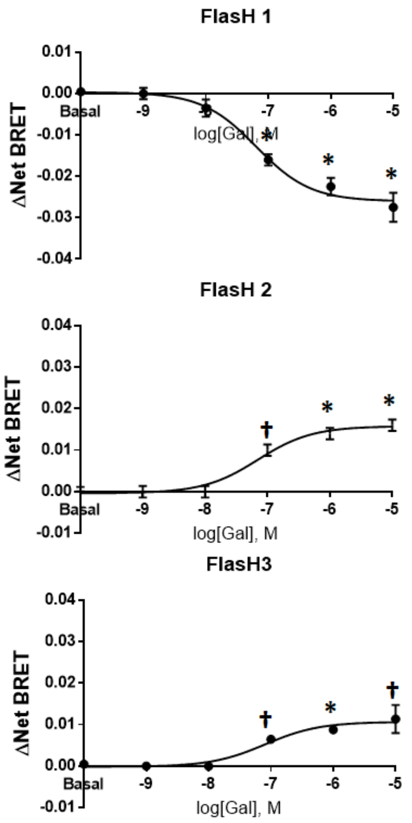

Supplementary Figure 5. β-arrestin2 intramolecular FlasH BRET signal to receptor occupancy. Ligand concentration-dependence of Galr2-induced changes in intramolecular FlasH BRET. HEK293 cells were co-transfected with plasmid cDNA encoding the Galr2 and the NLuc-β-arrestin2-FlasH1-3 biosensor. Stimulations were performed for 2 min using the indicated agonist concentration. The graph depicts the ΔNet BRET mean ± standard error (s.e.m.) values of three independent experiments performed in triplicate. In all panels †$P < 0.05$, *$P < 0.005$, greater or less than the vehicle stimulated control.

Supplementary Table 1. Primer sequences used to generate Nluc-β-arrestin2-FIAsH1-3.

Primer	Sequence
BamHI NanoLucF	CCGAGCTC*GGATCC*ACCATGGTCTTCACACTCGAAGATTTCG
NanoLucR	CCCGGGTTTCTCCCCCGCCAGAATGCGTTCGCACAGCCG
Beta arrestin 2F	GAACGCATTCTGGCGGGGGAGAAACCCGGGACCAGGGTC
Beta arrestin 2 EcoRI R	TATCTGCAGAATTCCTAGCAGAGTTGATCATCATA
FlasH 1aR	CAAAGTCTACGCCGCAACAACAACCAGGACAACAGGCCTTTCCTGTATCCTC
FlasH 1bF	GGATACAGGAAAGGCCTGTTGTCCTGGTTGTTGTTGCGGCGTAGACTTTGAG
FlasH 2aR	ATGTGGAGCTGGGAGAACAACAACCAGGACAACATACCTGGTCATCTTGTTC
FlasH 2bF	ACAAGATGACCAGGTATGTTGTCCTGGTTGTTGTTCTCCCAGCTCCACATTC
FlasH 3aR	TATCTGCAGAATTCTTAACAACAACCAGGACAACAGCAGAGTTGATCATCATAGTC
